# A Novel Approach to Generate a Virtual Population of Human Coronary Arteries for *In Silico* Clinical Trials of Stent Design

**DOI:** 10.1109/OJEMB.2021.3082328

**Published:** 2021-05-20

**Authors:** Dimitrios S. Pleouras, Antonis I. Sakellarios, George Rigas, Georgia Karanasiou, Panagiota Tsompou, Gianna Karanasiou, Vassiliki Kigka, Savvas Kyriakidis, Vasileios Pezoulas, George Gois, Nikolaos Tachos, Aidonis Ramos, Gualtiero Pelosi, Silvia Rocchiccioli, Lampros K. Michalis, Dimitrios I. Fotiadis

**Affiliations:** Department of Biomedical ResearchFORTH-IMBB GR 45110 Ioannina Greece; Department of Biomedical ResearchFORTH-IMBB GR 45110 Ioannina Greece; Unit of Medical Technology and Intelligent Information Systems, Department of Materials Science and EngineeringUniversity of Ioannina GR 45110 Greece; Unit of Medical Technology and Intelligent Information Systems, Department of Materials Science and EngineeringUniversity of Ioannina GR 45110 Greece; Department of Cardiology, Medical SchoolUniversity of Ioannina Ioannina GR 45110 Greece; Institute of Clinical PhysiologyNational Research Council 56124 Pisa Italy

**Keywords:** Cardiovascular virtual population, clinical data augmentation, plaque growth modeling, *in-silico* clinical trials

## Abstract

*Goal:* To develop a cardiovascular virtual population using statistical modeling and computational biomechanics. *Methods:* A clinical data augmentation algorithm is implemented to efficiently generate virtual clinical data using a real clinical dataset. An atherosclerotic plaque growth model is employed to 3D reconstructed coronary arterial segments to generate virtual coronary arterial geometries (geometrical data). Last, the combination of the virtual clinical and geometrical data is achieved using a methodology that allows for the generation of a realistic virtual population which can be used in *in silico* clinical trials. *Results:* The results show good agreement between real and virtual clinical data presenting a mean gof 0.1 ± 0.08. 400 virtual coronary arteries were generated, while the final virtual population includes 10,000 patients. *Conclusions:* The virtual arterial geometries are efficiently matched to the generated clinical data, both increasing and complementing the variability of the virtual population.

## I. Introduction

The design and development of new stents requires an assessment process to ensure their safety and efficacy. This procedure consists of three phases of clinical trials on humans after the *in vitro* analysis and the *in vivo* assessment in animal studies. Each subsequent phase requires a different number of patients to be enrolled to secure the efficacy and safety of the new stent. In recent years, computational modelling and simulation enable *in silico* clinical trials towards reducing, refining, and partially replacing the real clinical trials [1] with significant benefits, in terms of cost, increased safety, and reduced side effects for the patients. *In silico* clinical trials are achieved through the utilization of computational models usually in the form of a medical digital twin and their application to human data. However, the proper evaluation of the *in silico* stent model is affected by the availability of human arterial samples. Therefore, the lack of large number of patient populations and also the invariability among the enrolled patients enhanced the need of creating virtual patients.

The treatment of a stenosed artery requires the restoration of the blood flow in the lumen area, which is usually achieved through percutaneous coronary intervention (PCI), where the implant called stent, is positioned through a catheter at the stenosed region. In recent years, several studies were performed focusing on the development of computational models that enable the investigation of the stent performance effect on the arterial physiology [2]. Nonetheless, this evaluation requires accurate 3D geometries of human arteries. A cost-effective way to implement such a study, is to utilize data from virtual patients. Several research groups have focused on creating virtual populations. More specifically, Entelos created the Physiolab Platform which is used for drug clinical trials in drugs and includes data for the cardiovascular disease, without any 3D arterial geometries [3]. Another simulation tool, PopGen, allows the creation of a virtual population, which consists of realistic human anatomical and physiological data, but still, this is not a dedicated tool for the coronary anatomy [4]. To the best of our knowledge, currently, a dedicated virtual population that can be utilized for virtual stenting and the evaluation of new stent designs is not available.

In this work, a workflow for the generation of a virtual population which consists of human patients with cardiovascular disease including clinical data and coronary arterial geometries (arterial lumen, outer wall, plaques) is presented. The process for creating the virtual population is implemented using a four-level approach. First, the imaging dataset is used for the 3D reconstruction of the arterial geometries, while clinical data are collected from retrospective patients. In the second level, virtual clinical data are generated using a data augmentation algorithm, which is improved to efficiently replace incomplete data without affecting the statistics of the original population. In the third level, the reconstructed geometries are used as inputs in a computational model describing plaque growth which simulates the atherosclerotic plaque evolution and generates new virtual arterial geometries. At the fourth level, the results of the virtual clinical data with the virtual arterial geometries are combined to generate virtual patients, accompanied by clinical and geometrical arterial data. The novelty of this work regarding the virtual population is that it combines two different approaches for the generation of virtual patients: one for the clinical data generation and one for the arterial geometries. It is the only available population which includes coronary arteries to be used for simulation purposes. The developed virtual population is publicly available for research purposes at http://cardiovascularvirtualpopulation.eu.

## II. Materials and Methods

**Fig. 1.**
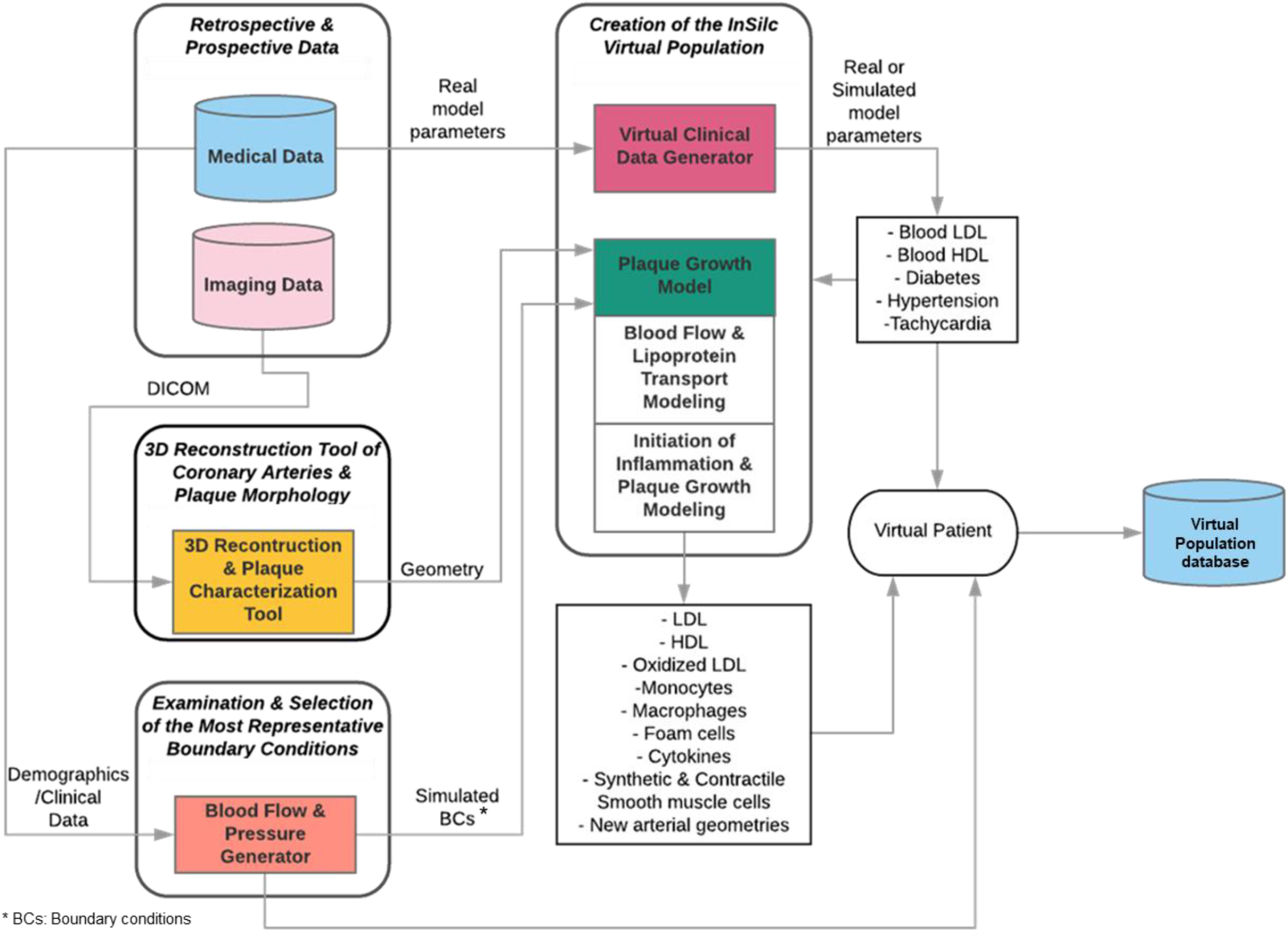
Information flow of the proposed virtual population generation.

The developed virtual population and virtual patients consist of realistic virtual geometries and virtual clinical data, respectively. The information flow of the proposed approach is presented in Fig. 1.

### A. Datasets and 3D Reconstruction

Human data are available from the SMARTool [6] (186 patients) and the InSilc project (50 patients). Clinical and CTCA imaging data are available from 186 patients which are used for the creation of the virtual clinical variables (Supplementary Table S1). The 3D reconstruction of these arteries was achieved using an already developed and validated methodology [7], [8]. From the InSilc dataset, IVUS and OCT and X-ray angiography data were used for the 3D reconstruction using established and validated methodologies [9]–[12]. All patients gave their written informed consent to participate in the study and the procedures followed were in accordance with institutional guidelines.

### B. Virtual Clinical Data Generation

The InSilc virtual clinical data generation is based on the statistical modeling of real data. Our approach is based on the joint multivariate distribution model. In general, each real patient's clinical data is assumed as a multivariate vector that consists of medical covariates and their corresponding values [35].

The objective of generating virtual patients with possible and realistic combinations of data is handled using the method of the total multivariate distribution of the clinical covariates. By using this approach, the distribution and the interrelationships of the real population covariates are evaluated in order to extract the multivariate distribution function, which is a function that describes the probability of a given combination of covariate values. All patient data, which are expressed as vectors of covariates, can be used to create a multivariate distribution, which can be represented as a multi-dimensional surface. Then, each patient vector represents a point of this surface. The function defining the surface of this distribution is statistically addressed as the joint multivariate function. More specifically, it describes the probability of a patient's covariate values to belong in the real population. Vectors with unrealistic values or unrealistic combinations of covariate values have zero probability.

Virtual patients are generated by a sampling technique that generates virtual multivariate vectors from surface of the multivariate distribution of the real dataset. By using an appropriate sampling technique, virtual patients are generated in such a way to present the same covariate interrelationships with the corresponding real patients. Using this approach, the possibility of generating unrealistic combinations of covariate values is implicitly eliminated.

#### 1) Multivariate Normal Distribution - Method of Joint Multivariate Function

This method assumes that the real population presents a multivariate normal distribution. In this case, the joint multivariate function *f(x)* is known and can be described by Equation (1), in which $x$ is the vector of covariates, *n* is the number of individuals of the real population, while $\mu $ is a vector of the covariate means (*μ_1_, μ_2_,..., μ_n_*)^T^, defined by Equation (2), and *Σ* is the covariance matrix (13,14).

\begin{align*}
f\left(x \right) &= {\left({2\pi } \right)^{ - n/2}}{\left| \Sigma \right|^{ - 1/2}}{e^{\left[ {\frac{{ - {{\left({x - \mu } \right)}^T}{\Sigma ^{ - 1}}\left({x - \mu } \right)}}{2}} \right]}}, \tag{1}\\
\mu &= \frac{1}{{n - 1}}\mathop \sum \limits_{i = 1}^n {x^{\left(i \right)}}. \tag{2}
\end{align*}

The joint multivariate function requires information about the correlation of any medical covariate pair. Specifically, this information is provided by the covariance of every covariate pair in the form of a matrix, the covariance matrix. In case of lack of the covariance values, the covariance matrix can be evaluated using Equation (3), where *Σ_ij_* is the covariance of *x_i_* and *x_j_*.

\begin{equation*}
{\Sigma _{ij}} = \frac{1}{{n - 1}}\mathop \sum \limits_{k = 1}^n \left({{{({x_i})}^{\left(k \right)}} - \overline {({x_i})} } \right)\left({{{({x_j})}^{\left(k \right)}} - \overline {({x_j})} } \right). \tag{3}
\end{equation*}

Given that the real population presents a multivariate normal distribution and the eigen-decomposition of the covariance matrix is possible, the generation of virtual patients is possible using the eigenvalue matrix *Λ* and the eigenvector matrix *U* of the covariance matrix, the vector of the covariate mean values *μ*, and *k* vectors of normal random values ${Z_i}$∊ $N({01})$, where *k* is number of the desired virtual patients (15). The algorithmic approach for this method is the following:

1) Evaluate *Λ* and *U* from the eigen-decomposition of *Σ.*

The decomposition of the covariance matrix *Σ* is performed using the Cholesky algorithm, which is an iterative algorithm that decomposes any symmetric positive definite matrix, such as the covariance matrix *Σ=UΛU^T^*.

1) Create the normal random vectors ${Z_i}$∊ $N({01})$.
2) For
  \begin{equation*}{\rm{For }}\ i = 1,2, \ldots,{\rm{ }}n \to nx_I^\prime = {u_i} + U{\Lambda ^{(1/2)}}{Z_i} \tag{4}
\end{equation*}

#### 2) Dealing with Categorical Values

The categorical values are matched to integer values allowing the extraction of the covariance matrix. However, simulation of virtual clinical data can result in non-integer, continuous values of the categorical covariates. The objective is to match the continuous values into integer ones, based on a continuous critical value (*CrV*) (16). According to this, any continuous value in the range of [*CrV 1, CrV 2*] is matched to the integer value belonging in this range of values. In this instance, any simulated continuous value in the range [0.5, 1.5] can be matched to the integer of value 1. Therefore, in the case of normal distributions, *CrV* can be evaluated as the average value of two sequential integers. However, in the case of log-normal distributions, *CrV* is given by Equation (5), where in the case of a categorical covariate *X* with *k* discrete values, *μ* =mean(ln(*x*)), *σ* =SD(ln(*x*)), *P_i_* is the proportion of subjects in the empirical distribution with categorical value *x_i_ (i ≤ k)*, and

\begin{equation*}
CrV\left({\mu,\sigma,\ {P_i}} \right) = \ {e^{\mu + \sigma \ NORMINV\left({{P_i}} \right)}} \tag{5}
\end{equation*}

In our case of log-normal distributions, the discretization of the covariate values is performed after the virtual data transformation from the log-normal system to the normal one. Therefore, the first method is implemented, which uses as a *CrV* the average value of two sequential integers.

#### 3) Restricting the Generation of Negative Values

All biological covariates are positive by definition. Therefore, a criterion that can be used is to remove all the virtual patients with any “faulty” negative values of the biological covariates. However, this could lead to severe distortion of the virtual multivariate distribution, which would not resemble the original one. Therefore, to efficiently constrain the simulated covariates from getting negative values, a common statistical technique was implemented, using the log-normal distributions for the simulation of medical covariates (17,18). According to this, the extraction of the joint multivariate distribution is performed using the logarithms of the real dataset values, while the final virtual clinical data result from the exponentiation of the simulated data (Fig. S1). However, the transformation of the normal distributed covariates to log normal distributed covariates requires the alteration of the second step of the algorithmic approach of the method of the joint multivariate function. More specifically, *Z* should now be a log-normal random vector belonging to the log-normally distribution (01) ${Z_i}$∊ $LN({01})$. Using this approach as a data filtration, virtual data are efficiently constrained to be positive.

#### Replacing the Missing Values of the Original Dataset

A novelty of this work is our approach of replacing any missing values of the real dataset with plausible values based on the inverse procedure of the algorithm used for the virtual clinical data generation. According to the previously described method of joint multivariate distribution, virtual clinical data result from Equation (4), utilizing the vector of the covariate means, the eigenvalue matrix *Λ* and the eigenvector matrix *U* of the covariance matrix, the vector of the covariate mean values *μ*, and a vector of log-normally distributed random values *Z ∊ LN(0, 1)*. To simplify our analysis, Equation (5) is transformed into Equation (6), using only the result of the multiplication of the eigenvalue matrix *Λ* and the eigenvector matrix *U* of the covariance matrix.

\begin{equation*}
x_i^{\prime} = {\mu _i} + U {\Lambda ^{1/2}} {Z_i} \mathop \Rightarrow \limits_{}^{K = U{\Lambda ^{1/2}}} x_i^{\prime} = {\mu _i} + \ K{Z_i} \tag{6}
\end{equation*}

In general, the original problem considered the $\ x_i^\prime$ as an unknown vector, while all others are known. In the case of the inverse problem, $ x_i^\prime$ is supposed to represent a real patient's clinical data, while all other variables are known except the vector$\ {Z_i}$, which is considered as an unknown vector. The rationale of our approach is that we can implement Equation (6), to evaluate a possible vector $\ {Z_i}$, which satisfies this equation and results to the half-known vector of $\ x_i^\prime$. In this instance, the resulted $\ {Z_i}$, can be used to define the remaining unknown values of $\ x_i^\prime$. Therefore, the equation that we need to solve is:

\begin{equation*}
\ x_i^\prime = {\mu _i} + \ K{Z_i}\mathop \Rightarrow \limits^{} {Z_i}\ = \ \frac{{\ x_i^\prime - {\mu _i}}}{K} \tag{7}
\end{equation*}

However, given that $\ x_i^\prime$ has several unknown values, the solution of Equation (7) can result in infinite $\ {Z_i}$ combinations. Any random selection of solution may result in a vector$\ Z$, which will seriously violate the condition of *Z* ∊ *LN(0, 1).* In this case, to result in a vector $\ Z$ with values that will be as close as possible in the range of the log-normal distribution *LN(01),* an optimization algorithm is used. In the case of equations with infinite solutions, as Equation (7), this algorithm regularizes the solution favoring the least norm (19). The norm of a vector is defined as the length or magnitude of the vector and can be calculated using Equation (8).

\begin{align*}
\left\| Z \right\| = \ \sqrt {{Z_1} + \ {Z_2} + \ {Z_3} + \ldots + \ {Z_n}}. \tag{8}
\end{align*}

### C. Virtual arterial Geometries Generation

Our approach is aiming to the utilization of a plaque growth model (20,21) which is used for the simulation of coronary arterial disease progress in human data with the potential to predict atheromatic areas of risk. The development of this model requires the proper definition of the equations that define the biologic processes responsible for atherosclerosis, using the state-of-the-art models, but also the latest available in literature experimental data. The necessary input for this model is the lumen domain and the arterial wall domain. Moreover, patient specific variables are used in the model, such as the serum LDL and HDL concentration as well as the pressure and the clinical risk factors of hypertension and diabetes. The simulation of each patient is used to extract arterial geometries at yearly time points. In this concept, for each reconstructed artery, four additional arterial geometries (representing the arterial geometry after 1, 2, 5 and 10 years) are generated.

The employed model has been previously validated and it can be used to simulate the plaque growth and lumen narrowing of 3D reconstructed coronary arteries (20). For this purpose, a multi-level approach is implemented. Initially, blood flow is simulated to calculate the endothelial shear stress (ESS), which is applied as input in the next level of the model. In particular, the main mechanisms of atherosclerosis are simulated using diffusion-convection-reaction equations. The mechanisms of LDL and HDL transport, LDL oxidation, inflammation triggering through the monocytes and macrophages accumulation, foam cells formation as well as the collagen formulation and the smooth muscle cells proliferation, are simulated. The plaque volume is estimated assuming that the plaque consists of smooth muscle cells, collagen and foam cells. The final level of the plaque growth model is to utilize the calculated plaque volume and its integration in a structural finite element analysis simulation to calculate the arterial wall deformation.

### D. Virtual population Generation

**Fig. 2.**
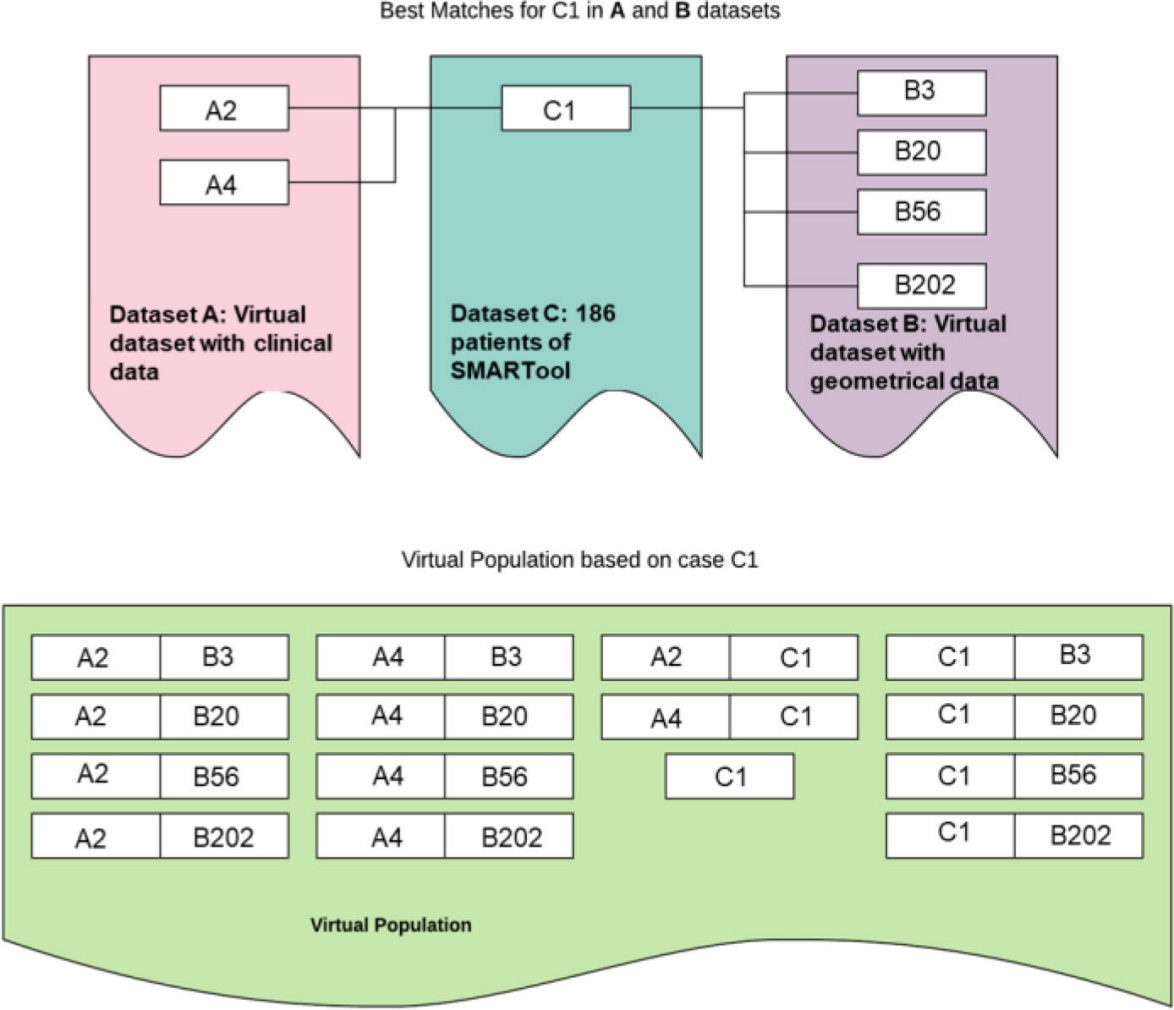
The generation method of virtual population using real datasets of clinical data and arterial geometries.

Through the utilization of the aforementioned methodologies, two virtual datasets are created: (i) one with clinical information, based on the statistical modeling approach and, (ii) one with imaging/morphological information, using the plaque growth modeling approach. The final virtual population combines the two datasets to generate virtual patients with clinical and geometry data. For the purpose of combining the two virtual datasets, we utilize the original SMARTool population of 186 patients which includes clinical and geometrical data. Thus, three different datasets have been utilized (i.e., the SMARTool dataset with all types of data, one virtual with geometrical reconstructed data and one virtual with clinical data only) (Table II) to create the final virtual population.For the creation of a large database of a virtual population, yet realistic patients, the concept of probabilistic record linkage (22), originally developed for matching two datasets, will be extended. This is an extension of the original concept of deterministic matching which relies on the exact match of specific fields. In previously developed algorithms its use was mainly for clinical or research purposes where the accuracy of matching is crucial. In virtual populations, on the other hand, a degree of “error” or “variance” is required to create a large variety of cases, keeping, however, all generated cases biologically valid. In our case we have three datasets, A, B and C; dataset C (186 patients from SMARTool population) includes all types of features from both A (virtual dataset with clinical information) and B (virtual dataset with geometrical information). Dataset C will be included (after de-identification) in the virtual population per se. To include the other two datasets (A and B) we need to merge them with C or together using C as a “linkage” table. The method is presented graphically in Fig. 2 and is outlined in the following steps:

• Define a matching probability threshold for A-C and B-C dataset matching, ${\hat{W}_{AC}}$ and ${\hat{W}_{BC}}$, respectively.
• Apply the probability recording linkage method on datasets A and C, and B and C. For each sample C, ${c_k}$ is the matching weight and the $w_{AC}^{ik}$ with sample A is estimated. The same applies for B and C where the matching weights $w_{BC}^{jk}$ are estimated for each sample of B.
• For each sample ${c_k}$ in the dataset C choose samples in A that have matching probability $w_{AC}^{ik}$ >${\hat{W}_{AC}}$, and create the set $V_{AC}^k$. Also, from dataset B take all samples ${b_k}$ where $w_{BC\ }^{jk} > {\hat{W}_{BC}}$, and create the set $V_{BC}^k$.
• The virtual population consists of dataset C, and all combinations of $V_{AC}^k\ $and $V_{BC}^k$ for each sample ${c_k}.$

## III. Results

A patient population of 186 patients (SMARTool project) was used by the previously described statistical model, to enable the extraction of a virtual population of 10000 patients. The generated virtual population is uploaded and available for research purposes at http://cardiovascularvirtualpopulation.eu. The Supplementary material presents some screenshots (Figs. S2-S4) from the developed database. The database integrates several functionalities such as creating a sub-population based on the applied filters or viewing the arterial geometries of the selected patient. To our knowledge, this is the largest population with clinical data and 3D coronary arterial geometries.

The virtual clinical data generation required less than a minute to be generated. However, to ensure the validity of the statistical model, several analyses were performed to compare the virtual with the real data. Initially, the mean averages of the virtual covariates were evaluated and compared to their corresponding realistic ones. We also used the evaluation metrics of Goodness of fit (gof) using the Kolmogorov–Smirnov test, the Pearson's product moment correlation coefficient, the mean, standard deviation, skewness and kurtosis.

Tables I and II present the Mean, Standard Deviation (SD), Skewness and Kurtosis for the real and virtual variables.

**TABLE 1:** Mean, Standard Deviation (SD), Skewness and Kurtosis for the Real and Virtual Variables

	Real values				virtual values				
	Mean	SD	Skewness	Kurtosis	Mean	SD	Skewness	Kurtosis	P-value
**Age**	60.78	8.09	-0.36	-0.25	60.38	7.25	-0.05	-0.55	0.197
**Body mass index (BMI)**	27.31	3.51	0.59	0.39	27.63	3.29	0.27	-0.20	0.02
**Systolic blood pressure**	138.99	16.92	0.20	0.59	138.29	16.36	0.25	-0.12	0.197
**Alanine aminotransferase**	19.83	9.59	1.58	3.16	20.64	8.42	1.08	1.36	0.002
**Alkaline phosphatase (U/l)**	49.47	18.35	0.86	0.61	52.01	17.13	0.74	0.35	0.027
**Aspartate aminotransferase (U/l)**	23.32	8.95	2.14	10.84	24.53	8.67	0.94	1.32	0.0001
**Gamma-glutamyl transferase (U/l)**	34.44	18.11	1.09	1.10	34.67	16.87	1.12	1.20	0.522
**Creatinine (mg/dl)**	0.85	0.19	0.28	-0.63	0.85	0.17	0.12	-0.36	0.654
**Uric acid (mg/dL)**	5.60	1.19	0.50	0.35	5.69	1.10	0.38	-0.07	0.014
**Glucose (mg/dL)**	105.50	23.97	2.99	16.24	107.01	19.77	0.67	0.61	0.0001
**Triglycerides (mg/dL)**	112.79	56.85	1.23	1.63	115.34	52.96	1.00	0.86	0.27
**Total Cholesterol (mg/dL)**	190.60	49.34	0.28	-0.50	191.71	46.51	0.47	-0.20	0.984
**Low-density lipoprotein (LDL) (mg/dL)**	114.09	42.26	0.36	-0.27	115.22	42.63	0.73	0.21	0.612
**High-density lipoprotein (HDL) (mg/dL)**	54.27	17.31	1.32	4.63	53.63	16.32	0.92	1.26	0.137
**Hs-C Reactive Protein (mg/dL)**	0.37	0.68	5.25	36.14	0.51	0.37	1.35	3.48	0.0001
**Interleukin6**	1.07	1.29	3.21	13.87	1.34	0.91	1.62	4.28	0.0001
**Leptin**	9.69	8.74	1.88	5.26	8.83	7.86	2.07	5.55	0.769
**ICAM1**	197.59	74.11	1.75	4.16	208.86	66.73	0.85	0.84	0.0001
**VCAM1**	525.35	120.58	1.57	3.77	537.94	107.03	0.79	0.82	0.0001
**hs-cardiac Troponin T**	9.23	29.96	11.00	123.11	8.33	4.91	2.11	8.03	0.0001
**Insulin (mIU/L)**	9.90	9.14	3.43	15.75	9.91	6.82	1.99	6.88	0.001
**MMP2 protein plasma (ng/L)**	171.28	56.21	-0.04	-0.67	158.51	50.88	0.51	-0.32	0.0001
**MMP9 protein plasma (ng/L)**	130.05	172.60	2.46	6.46	130.82	137.34	2.50	7.67	0.0001

**TABLE 2:** Percentage Distribution of the Real and Virtual Categorical Variables

	Real values		Real values with filled missing values		virtual values	
	Yes (%)	No (%)	Yes (%)	No (%)	Yes (%)	No (%)
**Gender (Yes=male)**	56.99	43.01	56.99	43.01	61.45	38.55
**Family History**	49.46	50.54	48.92	51.08	45.06	54.94
**Current Smoking**	20.97	79.03	12.90	87.10	12.36	87.64
**Past Smoking**	45.70	54.30	38.71	61.29	37.72	62.28
**Diabetes Mellitus**	16.13	83.87	14.52	85.48	15.06	84.94
**Dyslipidemia**	69.35	30.65	69.35	30.65	61.55	38.45
**Hypertension**	63.98	36.02	61.29	38.71	58.74	41.26
**Metabolic Syndrome**	0.54	99.46	0.54	99.46	0.00	100.00
**Obesity**	17.74	82.26	17.74	82.26	16.63	83.37
**Statins**	46.77	53.23	45.70	54.30	41.94	58.06

Fig. S5 (vertical axis represent the number of patients) present the gof and the distribution of some indicative variables, while Table III presents the gof for all generated variables. It is clear, that good agreement is observed for almost all variables in which gof is < 0.2. Regarding the correlation between the real matrix of variables with the virtual one, we found an average coefficient 77.54 and 80.88 and SD = 119.95 and 125 for the real and virtual data, respectively.

**TABLE 3:** GOF Values for All Variables, Mean and Standard Deviation

Variable	GOF	Variable	GOF
**Age**	0.079	**Gamma-glutamyl transferase (U/l)**	0.059
**Gender**	0.062	**Creatinine (mg/dl)**	0.053
**Family History**	0.039	**Uric acid (mg/dL)**	0.116
**Current Smoking**	0.006	**Glucose (mg/dl)**	0.157
**Past Smoking**	0.016	**Triglycerides (mg/dL)**	0.073
**Diabetes Mellitus**	0.005	**Total Cholesterol (mg/dL)**	0.033
**Dyslipidemia**	0.078	**LDL (mg/dL)**	0.055
**Hypertension**	0.026	**HDL (mg/dL)**	0.085
**Metabolic Syndrome**	0.005	**Hs-C Reactive Protein (mg/L)**	0.351
**Obesity**	0.011	**Interleukin6**	0.294
**BMI**	0.111	**Leptin**	0.048
**sbp**	0.079	**ICAM1**	0.178
**Current Symptoms**	0.104	**VCAM1**	0.178
**Statins**	0.038	**hs-cardiac Troponin T**	0.220
**Alanine aminotransferase**	0.138	**Insulin (mIU/L)**	0.142
**Alkaline phosphatase (U/l)**	0.108	**MMP2 protein plasma (ng/L)**	0.190
**Aspartate aminotransferase (U/l)**	0.164	**MMP9 protein plasma (ng/L)**	0.152

**Fig. 3.**
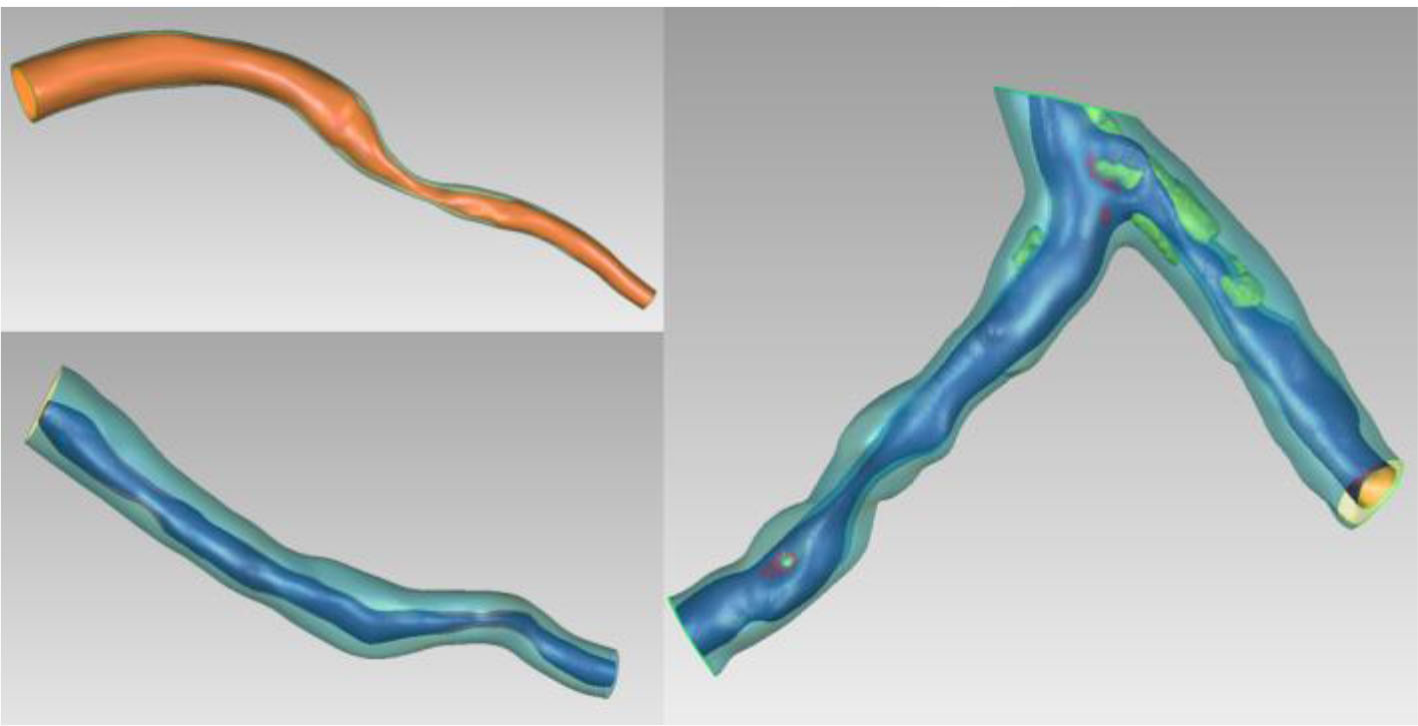
Case examples of coronary arteries. When CTCA was used, bifurcations were also enabled. Also, when plaques are present, they were reconstructed as well (yellow objects on the bifurcated artery)..

Plaque growth modelling was applied to 100 patients from the SMARTool population where clinical, molecular and CTCA imaging data were available. This enabled the generation of 400 virtual arterial geometries since we extract the geometries at 1, 2, 5 years, and the last time point of the simulation. Additionally, the dataset of the virtual arterial geometries included 50 arteries which have been reconstructed using the OCT/IVUS and X-ray angiography. Some examples of the baseline arterial geometry and the new one at the final time point presented in Fig. 3. The final step of our methodology includes the combination of the 10000 virtual clinical data with the 550 (100 real patients from the SMARTool population, 400 virtual arteries from the plaque growth model and 50 reconstructed arteries using OCT/IVUS and X-ray angiography) virtual arterial geometries. Using this approach, a virtual population of patients with clinical and arterial geometries data has been created. Moreover, this population includes only patients with >50% stenosis to be used in *in silico* clinical trials in the stent industry.

## IV. Discussion

The generation of the InSilc virtual population includes several conceptual novelties, as well as innovative implementations and methodologies. To the best of our knowledge, this is the first attempt to integrate 3D arterial information and clinical information towards the creation of a virtual population of human coronary arteries. More specifically, the generated virtual population combines information from two methodological levels: (i) a statistical model of clinical data augmentation, and (ii) a computational model of plaque growth. In brief, regarding the clinical data augmentation, we employ a statistical modeling approach using a real dataset of 186 patients, implementing a new approach for the imputation of the missing values. Using this methodology, we achieved the creation of 10000 virtual patients with clinical information, which is in very good agreement with the real cases. Concerning the plaque growth, our work has been based on an already available computational plaque growth model. It is the first time, in which a plaque growth model generates and delivers as output realistic 3D arterial geometries. This virtual population is the only one available in the literature which can be used for *in silico* clinical trials for coronary stents.

The generation of virtual populations based on the data augmentation of existing datasets is gaining popularity over the last years. An example of a virtual population generation mechanism is the Synthea [23] which simulated the data of an electronic health record system using the basic demographics of the Massachusetts population and based on specific disease models. Similar efforts have been employed to create virtual patients with specific measurements (i.e., glucose measurements) using mathematical models [24] or using very specific features [25] (non-demographics). The methodology proposed for our virtual population creation has similarities with the concept of Synthea, however, in our approach, a computational model to create additional virtual geometries has been also incorporated in our methodology. In brief, a dataset of virtual patients is composed based on real data (demographics, lab examinations and geometries) and then using the patient specific disease progression model, different arterial models under discussion can be generated.

Nowadays, *in silico* clinical trials are performed by complex system models, which are built using real patient data [13]. In the case when a large patient dataset exists, clinical trial simulations require the selection of a small representative sample of the original dataset. Opposing to the existence of large patient datasets, cases of limited datasets urge the need for the collection of further patient data, which can be an expensive even an impossible procedure. Therefore, several techniques, enabling data augmentation by introducing virtual patients in the original dataset, have been developed. This technique is considered reliable when applied to a dataset presenting non-Gaussian distributions of its covariates [26].

In our case of statistical modeling, the selected method of the joint multivariate function is a common and efficient method to introduce virtual patients in the limited original population of our study (186 real patients). However, this method can result exclusively in normal distributed covariates, which is in line with the current literature [17], [27]. Using the above-mentioned statistical modeling approach, we generated a virtual population, which consists of 10000 virtual patients with clinical data starting from a real population of 186 patients. The comparison between the real and virtual populations shows a good agreement in the distribution of variables. However, few variables represented not very satisfactory results (approximate 10% difference). This limitation mainly arises from the missing values for these variables.

The creation of this virtual database is part of the InSilc platform [5], a dedicated *in silico* cloud platform used to perform *in silico* clinical trials for the design, assessment and optimization of stents. The InSilc platform includes five multi-disciplinary and multi-scale models that simulate the performance of the scaffold in terms of deployment, drug-release, fluid dynamics and degradation while providing indications on the Myocardial Perfusion. Virtual patients are included in several scenarios of use, specially designed towards fulfilling the requirements of the different stakeholders of the InSilc platform, which are the stent industry, the interventional cardiologists, the Contract research organizations (CROs) and the research community. The following scenarios of use have been implemented: (i) Comparison of the performance of existing stents in the same virtual population, (ii) Comparison of stent performance in different anatomy configurations and patient conditions, (iii) Comparison of stent performance in different clinical procedures, and (iv) Design entirely new stents.

The importance of generating a virtual population in the field of virtual stent deployment is significant. Cardiovascular disease has a significant economic impact estimated to cost the EU economy 210 billion euro per year [28]. The global coronary stent market size is estimated at approximately 8 billion euro in 2019 [29]. The Cardiovascular Biomedical Industry invests in the development of new stents, however, the time-consuming development processes (3 to 7 years are required on average for bringing these devices to the market), and the associated high costs of a clinical trial (estimated between 31 and 94 million euro) are a bottleneck. On the other side, there are several issues with the clinical investigation of stents including: (i) *Inadequacy to demonstrate efficacy or safety*. Coronary stents clinical trial failures occur frequently in the last 10% of the pipeline where 90% of the activity needed to get the device out to market takes place. (ii) *Difficulties in patient enrolment.* Patient inclusion/exclusion criteria should result in a population that matches statistically the intended general patient population; however, study designers must account for additional concerns, including whether or not particular segments of a target population may have several comorbidities, leading to an additional higher risk of withdrawal and adverse events. (iii*) One size fits all issue.* The clinical trial designs essentially do not take into account the patient variability and complexities, and the heterogeneity of the enrolled in clinical trial patients are being translated into a similar heterogeneity of responses to stent implantation. The availability of a virtual population applied in the context of new stent design will increase patients’ safety, reduce clinical trials costs and as the final outcome will improve the clinical practice offering highly efficient stents with reduced side effects. FDA has recognised the benefits of modernizing real clinical trials and has already begun exploring the utilisation of *in silico* technology and virtual population to improve clinical trials. This strategy has been communicated through the 21st Century Cures Act that focuses on accelerating medical product development and bring innovations and advances to patients as well as through the creation of the Technology Modernization Action Plan (TMAP) with the mission to bridge the gap between scientific and technology advances [30], [31]. Concluding, the developed virtual population can potentially be used to replace or reduce the pre-clinical and animal studies, as well as to use a lower number of human patients with the same, however, power of the final outcomes [32].

## V. Conclusion

In this work, a novel concept for the generation of a virtual population of cardiovascular disease patients with virtual clinical and arterial geometrical data has been introduced. For this purpose, an approach for the statistical generation of virtual clinical data has been implemented, while a novel method was developed to treat efficiently incomplete data. Also, for the first time, a computational model has been used to reproduce virtual arterial geometries. Finally, the results of the two models have been combined to generate a unique virtual population of human patients with coronary arterial information adequate for *in silico* clinical trials in the stent industry.
